# Case report: Deterioration of infantile hemangioma related to oral or nebulized administration of β2-AR agonist: Three cases reports

**DOI:** 10.3389/fonc.2022.1000099

**Published:** 2022-11-10

**Authors:** Qiang Chen, Yunxuan Zhang, Chenyu Sun, Li Liu, Xiaoyan Luo, Hua Wang, Sili Ni

**Affiliations:** ^1^ Department of Dermatology, Children’s Hospital of Chongqing Medical University, National Clinical Research Center for Child Health and Disorders, Ministry of Education Key Laboratory of Child Development and Disorders, Chongqing Key Laboratory of Child Infection and Immunity, Chongqing, China; ^2^ Department of Pediatrics, Chongqing University Three Gorges Hospital, Chongqing, China; ^3^ Department of Burn and Plastic Surgery, Children’s Hospital of Chongqing Medical University, Chongqing, China; ^4^ AMITA Health Saint Joseph Hospital Chicago, University of Illinois at Chicago, Chicago, IL, United States

**Keywords:** infantile hemangioma, recurrence, propranolol, β2-adrenergic receptor, salbutamol, procaterol

## Abstract

Infantile hemangioma (IH) is a benign vascular tumor, characterized by a unique sequence of non-linear growth and spontaneous involution. Some hemangiomas require intensive treatment to avoid functional and aesthetic insufficiency. Although β-adrenergic receptor (β-AR) antagonists have been increasingly used as the first-line treatment since 2008, the IH rebound still exists with uncertain mechanism. Here, we report three cases of abrupt IH deteriorations that are mainly related to β2-AR agonist administration. Potential IH proliferation induced by β2-AR agonists, especially from oral or nebulized approaches, should be recognized more widely by healthcare providers. Additionally, it is necessary to carry out large sample studies to analyze the influence of β2-AR agonist administration on the deterioration of IH.

## Introduction

Infantile hemangioma (IH) occurs in approximately 5%–10% of infants (1, 2). The potential risk factors for IH are prematurity, low birth weight, female gender, Caucasian, multiple pregnancy, progesterone therapy, and family heredity. Characterized by proliferative phase, plateau, and regression phase, the growth cycle of IH is non-linear but usually rapid during the first 3 months (3). Even though 90% of IH eventually involutes within 1–10 years, it still causes alarm about affecting aesthetic appearance and psychological development, as well as developing life-threatening complications if associated with vital functions (4–7). Oral propranolol therapy (OPT) is widely recognized for its efficacy and safety. Several mechanisms may involve in the treatment of IH, including vasoconstriction (8), angiogenesis inhibition (9, 10), and induction of apoptosis in endothelial cells (8, 11). However, rebound growth after propranolol discontinuation has been noted in 6%–25% of patients, and the exact mechanism remains unclear, which may be interfering with the age of discontinuation, deep IH component, and female gender (12–14).

In this study, we report three cases of increased size and redness in regressed and almost regressed IHs, which were mainly related to oral or nebulized administration of β2-adrenergic receptor (β2-AR) agonists. To the best of our knowledge, it is rarely reported and not widely recognized by healthcare providers (15). Clinical findings and laboratory evidence are articulated to emphasize the significance of clarifying the relationship between β2-AR agonists and IH deterioration in its management.

## Case presentation

### Case 1

**Figure 1:**
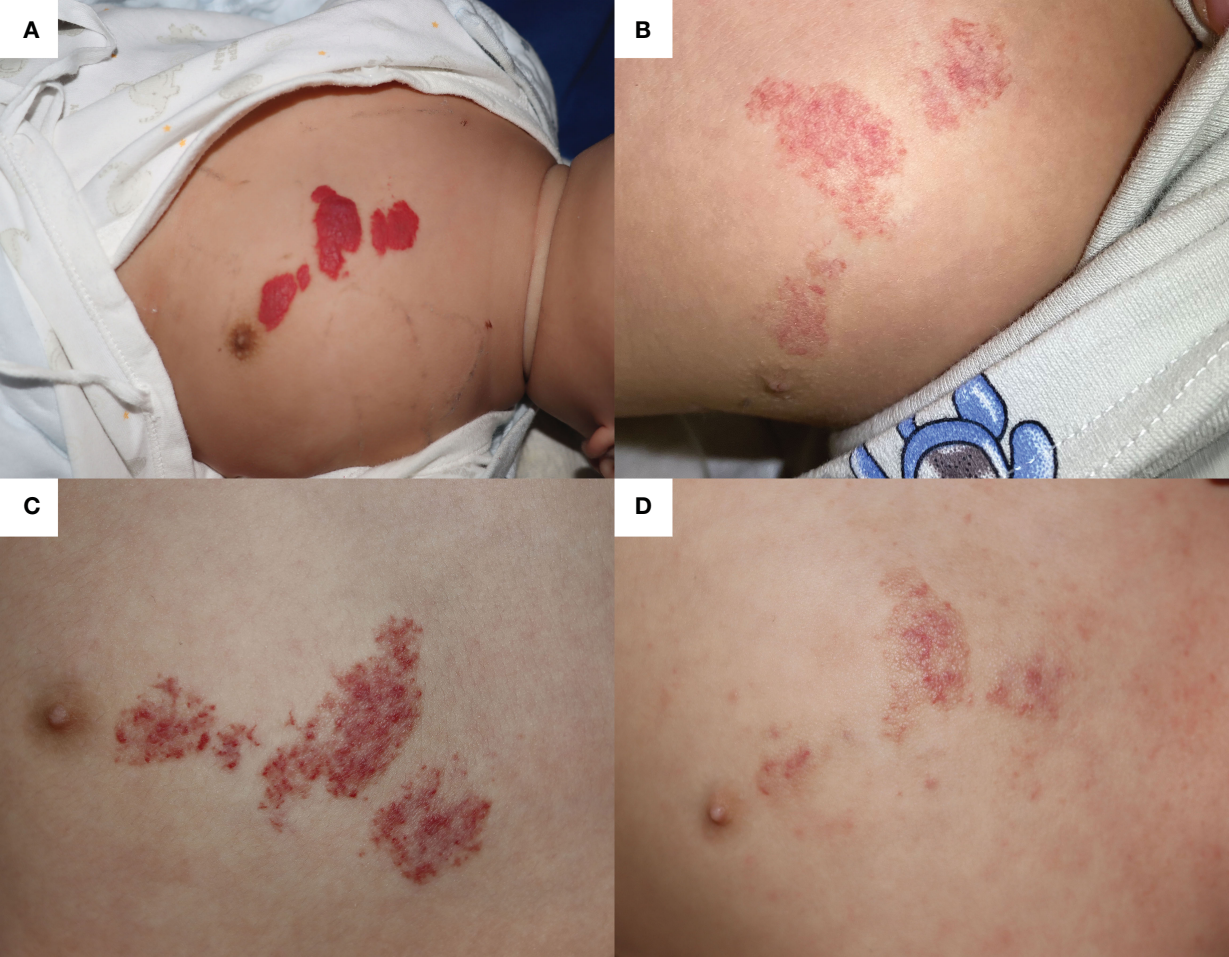
The infantile hemangioma in patient 1 at different periods. **(A)** Proliferating infantile hemangioma before oral propranolol therapy. **(B)** Involuted infantile hemangioma when the patient was admitted for pneumonia at the age of 13 months. **(C)** Infantile hemangioma worsened after nearly 1 day of oral procaterol therapy. **(D)** Involuted hemangioma after 1 month of oral propranolol therapy and one laser therapy.

A 5-month-old boy was diagnosed with IH and admitted to the Department of Dermatology at Children’ Hospital of Chongqing Medical University for primary OPT. He was a premature infant at 30 weeks gestation with a birth weight of 1.35 kg. The IH had been rapidly growing on his left chest wall since birth, measuring 3.0 × 1.5cm at maximum and 0.6 × 0.4cm at minimum with bright red color (**Figure 1A**). With close monitoring of vital signs, propranolol was initiated at 1.0 mg/kg per day and maintained at 2.0 mg/kg per day (increase by 0.5 mg/kg every 2 days). After discharge, propranolol was maintained at 2.0 mg/kg per day and had significantly improved the IH until this admission for pneumonia (**Figure 1B**).

At the age of 13 months, the patient suffered from severe pneumonia and received 3 days of non-invasive ventilation, antibiotics, and antitussive–expectorant nebulization inhalation (budesonide, ipratropium bromide, and ambroxol), during which OPT was discontinued to avoid bronchospasm. OPT was restarted as respiratory symptoms improved. Unfortunately, because of worsening bronchitis, OPT only lasted for 6 days and was replaced by topical timolol. Additionally, prednisone acetate tablets (7.5 mg twice a day) and the procaterol hydrochloride oral solution (2.5 ml twice a day) were added to relieve bronchospasm symptoms. After nearly 1 day of comprehensive treatment, the color of the IH progressed into deep red and showed a trend of proliferation (**Figure 1C**). However, because of the current prioritization of bronchitis treatment, more aggressive IH management could not be implemented. Finally, the vasodilation of IH was in significant remission in 1 month of OPT and a single 585-nm pulsed dye laser (PDL; Cynergy™, Cynosure^®^) therapy after discharge (**Figure 1D**).

### Case 2

**Figure 2:**
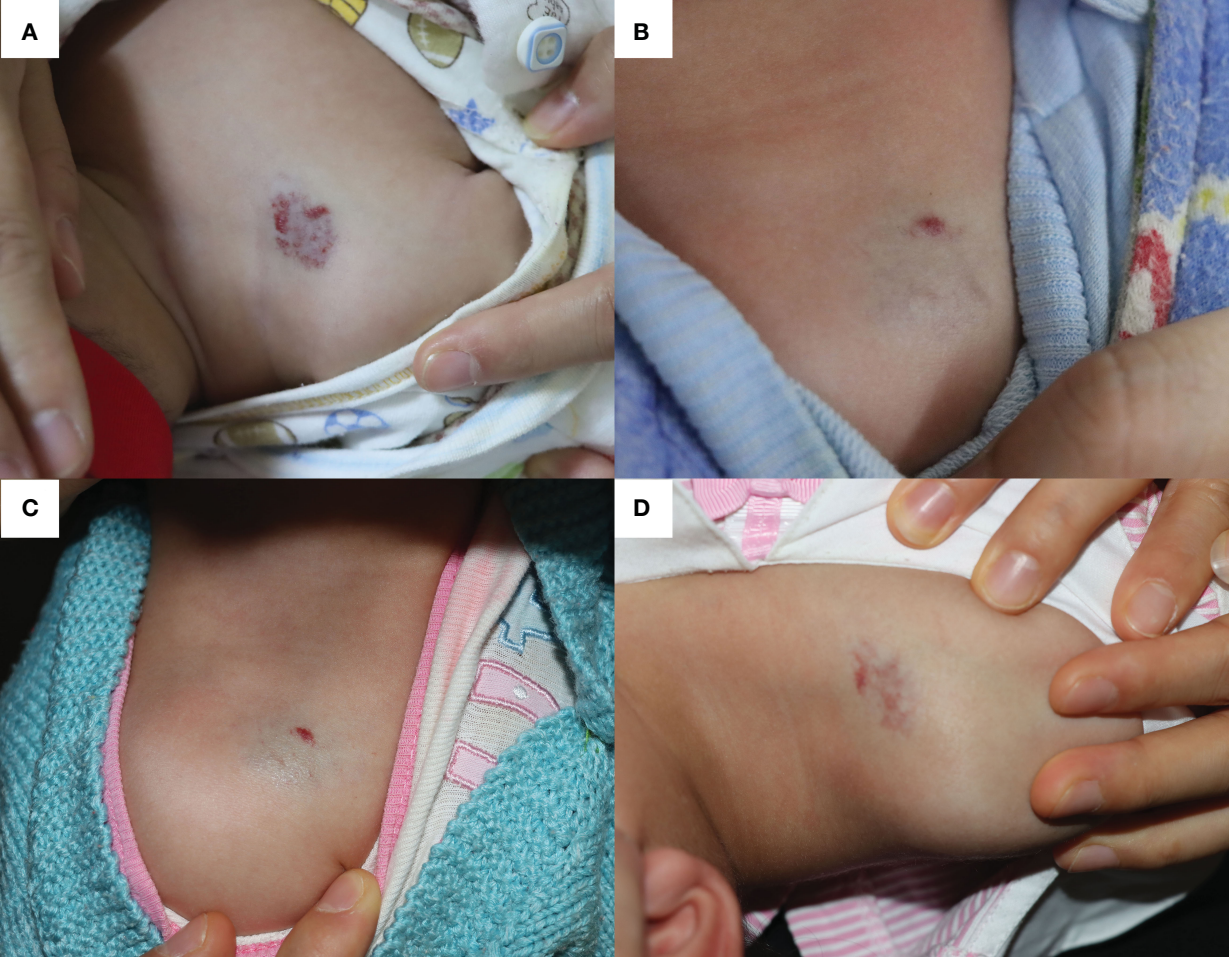
The infantile hemangioma in patient 2 at different periods. **(A)** Proliferating infantile hemangioma before oral propranolol therapy and laser therapy. **(B)** Involuted infantile hemangioma when the patient was admitted for acute bronchitis at the age of 15 months. **(C)** Infantile hemangioma showed a proliferation trend after 7 days of nebulized salbutamol treatment. **(D)** Infantile hemangioma worsened after receiving another 3 days of nebulized salbutamol therapy in the outpatient.

A 4-month-old boy was diagnosed with IH (located on the medial side of the right shoulder joint) and admitted for OPT (**Figure 2A**). With close monitoring of heart rate and blood pressure, propranolol was initiated at 1.0 mg/kg per day and gradually increased to 2.0 mg/kg per day during the 4 days of hospitalization (increase by 0.5 mg/kg every 2 days). After discharge, in addition to maintenance OPT at a dosage of 2.0 mg/kg per day, the patient also received five times of 585-nm PDL therapies, which helped to achieve significant and nearly complete involution of the IH (**Figure 2B**).

At 15 months old, he presented with acute bronchitis and started to receive nebulized inhalation treatment (salbutamol 2.5 mg twice a day and budesonide 1.0 mg twice a day). Meanwhile, OPT was replaced with topical timolol to avoid its bronchospasmodic effects. However, after 7 days of respiratory management, the IH showed a trend of proliferation (**Figure 2C**). As the respiratory symptoms did not completely resolve even after discharge, he received another 3 days of nebulized therapy in outpatient clinic, and the IH size further expanded (**Figure 2D**).

### Case 3

**Figure 3:**
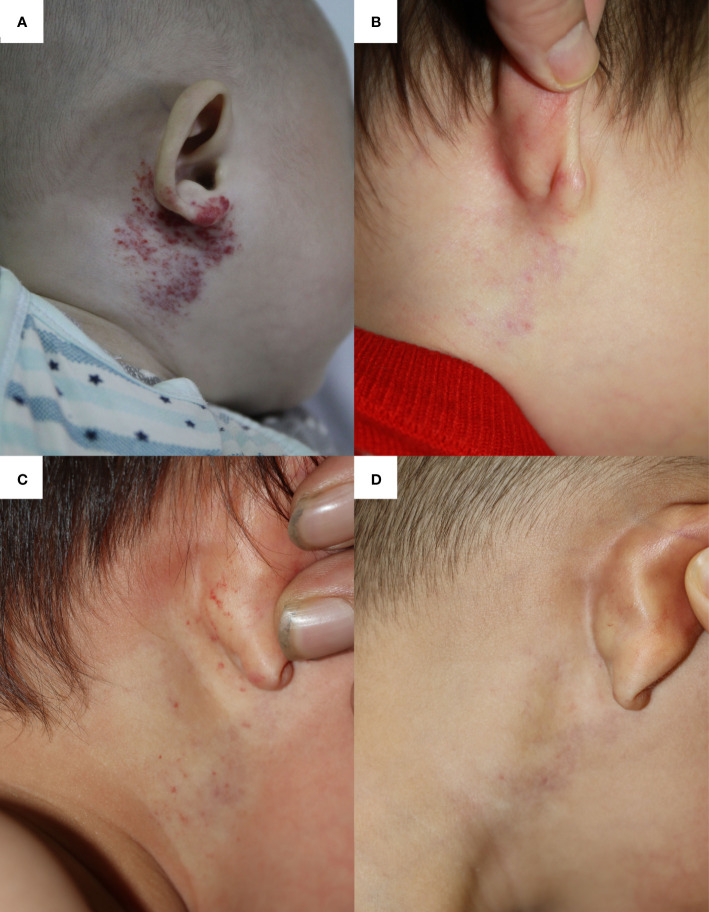
The infantile hemangioma in patient 3 at different periods. **(A)** Proliferating infantile hemangioma before oral propranolol therapy and laser therapy. **(B)** Involuted infantile hemangioma when the patient had completed the 10-month oral propranolol therapy and nine times of laser therapy at the age of 14 months. **(C)** Deterioration of infantile hemangioma after receiving 6 days of oral clenbuterol and nebulized salbutamol. **(D)** Involuted hemangioma after 1 month of oral propranolol therapy and one laser treatment.

A 4-month-old boy was diagnosed with IH, which was located on the right periauricular area (**Figure 3A**). Ten months of OPT (2.0 mg/kg per day) and nine times of 585-nm PDL therapies significantly reduced its size and faded its color into pale pink when he was 14 months old (**Figure 3B**). After evaluation, propranolol and 585-nm PDL therapy were discontinued as the residuum was simply left to fade over time. Fourteen months after OPT discontinuation, he suffered from acute bronchopneumonia and received antibiotics, clenbuterol hydrochloride oral solution (7.5 ml bid), and antitussive–expectorant nebulization inhalation (ipratropium bromide, budesonide, salbutamol) at a local clinic. After 6 days of respiratory management, increased redness and macules were found on the original IH (**Figure 3C**). Therefore, laser therapy and OPT were restarted. These newly developed hemangiomas faded rapidly after 1 month of comprehensive treatments (**Figure 3D**).

## Discussion

OPT is currently the first-line treatment for IH because of its high response rate and safety (16–20). It usually lasts for 6–12 months or longer to overcome the rapid growth during the proliferative phase (21). In most cases, a rapid effect can be observed on color and texture after several days of OPT at a dose of 2–3 mg/kg per day. Complete or nearly complete regression can be observed in 60% of cases after 6 months of treatment (16, 22). The mechanism of action may be associated with endothelial cells and pericytes (8, 23). As a non-selective β-AR antagonist, propranolol can inhibit the NO release induced by norepinephrine through the PI3K/Akt/eNOS/NO pathway, thereby inhibiting vasodilation (24). Propranolol can also enhance the expression of α-smooth muscle actin in pericytes by blocking the β2-AR pathway, which results in vasoconstriction (23, 25).

According to published literature, rebound growth was recorded in 6%–25% of IH cases after discontinuation of propranolol (12–14). In this study, we report dramatic IH involution after 8–10 months of OPT (discontinued or not) and abrupt deterioration during the treatment of bronchitis. For case 1, IH reproliferation only occurred 1 day within the second propranolol withdrawal but did not occur within the 3-day period of the first withdrawal. It suggested that propranolol withdrawal might not be the main cause of IH deterioration in this patient. Then, what if it was because of the inhalation of budesonide, ipratropium bromide, and ambroxol during the first withdrawal period? Budesonide is a locally acting glucocorticoid used mainly to reduce the contractile response of bronchial smooth muscle. Ipratropium bromide is a muscarinic receptor antagonist that has not been reported to be associated with IH or its pathway. Ambroxol is a typical expectorant medication that mainly reduces the viscosity of sputum. Additionally, even though there are potential pathways for these medications to cause IH deterioration, the symptoms should have appeared earlier. Therefore, we excluded the speculation of these medications and turned our attention to the specific course of orally taken procaterol hydrochloride, which showed a close sequence with deterioration and might suggest a causal relationship. For case 2, the nearly involuted IH showed progressive vasodilatation during a total of 10 days of propranolol withdrawal and nebulized salbutamol therapy. Because of the lack of further examinations, there was no direct evidence to definitively determine whether propranolol withdrawal or nebulized salbutamol therapy caused the deterioration in this case. Current reports of IH deterioration after propranolol discontinuation or β2-AR agonist administration suggested that it was not a common occurrence and the interval time varied widely from 1 to 8 months (12, 26). A variety of factors can play a role in IH deterioration, and the exact mechanism remains unclear. By analyzing the unique characteristics of each patient’s disease course, we may be able to identify some evidence to help determine the most reasonable interpretation. Shinji et al. (26) reported three cases of IH rebound in 1 month after completing the treatments, in multiple times. Nicole et al. (15) reported a case with a single course of IH rebound directly associated with intravenous salbutamol. Two important differences between these two studies were the frequency of IH rebound (multiple or single) and the external intervention such as the intravenous β2-AR agonists. Therefore, these two studies proposed interpretations for the recurrence in different considerations, the gene mutation of CYP2D6 (a cytochrome P450 isozymes) or the reaction of β2-AR agonists. For our patient, the single deterioration course and a clear history of salbutamol inhalation supported the use of β2-AR agonists as the culprit in a particularly high probability. For case 3, after 14 months of propranolol discontinuation, the involuted IH showed proliferation after 6 days of oral clenbuterol and nebulized salbutamol inhalation. The discussion above for patient 2 applies equally to this patient, and because the involuted IH had stopped growing for more than 1 year, the probability of relapse caused by propranolol withdrawal was extremely low at this point, and it would be more reasonable (than in the previous two cases) to believe that the deterioration was due to β2-AR agonist intake. Salbutamol, procaterol, and clenbuterol are common selective β2-AR agonists that produce opposite effects to β2-AR antagonist *via* the coincident intracellular β2-AR–driven proangiogenic pathway, such as promoting norepinephrine-induced NO release and inhibiting the α-smooth muscle actin expression in pericytes. Their combined effects enhance the vasodilation of IHs, which results in increased size and redness in clinical observations. Furthermore, a recent study showed no significant difference in β2-AR expression among proliferative, involutional, and propranolol-responsive hemangiomas, which may explain the role of β2-AR agonists in the involutional phase (12–14, 27). Therefore, we speculated that the aggravation of IH in these three patients was mainly due to β2-AR agonist administration.

Even though it is not the first report on IH deterioration associated with β2-AR agonist, it is still not taken seriously by most clinicians. Nicole et al. (15) reported a patient who developed overt IH rebound within 24 h of continuous intravenous salbutamol (2.0 mg/kg per min). It rebounded more quickly and profoundly than ours, possibly because the continuous intravenous administration achieved higher blood concentration of β2-AR agonist than our cases with oral or nebulized approach. This work complements the research on IH deterioration associated with the administration of β2-AR agonists and calls for special attention to their oral or nebulized approaches, as they are frequently prescribed in the treatment of pediatric respiratory diseases. Large-sample-size studies should be carried out to analyze the influence of β2-AR agonists on IH deterioration/rebound. Although whether β2-AR agonist is a risk factor still needs further evidence, current reports and evidence urge the healthcare providers to be more prudent when administrating β2-AR agonists to pediatric patients with IH. If β2-AR agonist cannot be avoided in some cases, patients and/or their legal guardians should be warned of the possible deterioration of IH.

## Data availability statement

The original contributions presented in the study are included in the article/Supplementary Material. Further inquiries can be directed to the corresponding author.

## Ethics statement

The studies involving human participants were reviewed and approved by Ethics Committee of Children’s Hospital of Chongqing Medica University. Written informed consent from the participants’ legal guardian/next of kin was not required to participate in this study in accordance with the national legislation and the institutional requirements.

## Author contributions

Conception and design: QC, YZ, and SN. Administrative support: SN and HW. Provision of study materials or patients: QC, LL, XL, and HW. Collection and assembly of data: YZ and CS. Data analysis and interpretation: YZ, QC, and CS. Native English polish and provided critical opinion: CS. Manuscript writing: All authors. Final approval of manuscript: All authors.

## Conflict of interest

The authors declare that the research was conducted in the absence of any commercial or financial relationships that could be construed as a potential conflict of interest.

## Publisher’s note

All claims expressed in this article are solely those of the authors and do not necessarily represent those of their affiliated organizations, or those of the publisher, the editors and the reviewers. Any product that may be evaluated in this article, or claim that may be made by its manufacturer, is not guaranteed or endorsed by the publisher.
