# Corrigendum: Case report: deterioration of infantile hemangioma related to oral or nebulized administration of β2-AR agonist: three cases reports

**DOI:** 10.3389/fonc.2023.1282607

**Published:** 2023-09-08

**Authors:** Qiang Chen, Yunxuan Zhang, Chenyu Sun, Li Liu, Xiaoyan Luo, Hua Wang, Sili Ni

**Affiliations:** ^1^ Department of Dermatology, Children’s Hospital of Chongqing Medical University, National Clinical Research Center for Child Health and Disorders, Ministry of Education Key Laboratory of Child Development and Disorders, Chongqing Key Laboratory of Child Infection and Immunity, Chongqing, China; ^2^ Department of Pediatrics, Chongqing University Three Gorges Hospital, Chongqing, China; ^3^ Department of Burn and Plastic Surgery, Children’s Hospital of Chongqing Medical University, Chongqing, China; ^4^ AMITA Health Saint Joseph Hospital Chicago, University of Illinois at Chicago, Chicago, IL, United States

**Keywords:** infantile hemangioma, recurrence, propranolol, β2-adrenergic receptor, salbutamol, procaterol

**Incorrect Affiliation**

In the published article, there was an error in affiliation 1. Instead of “Department of Dermatology, Children’s Hospital of Chongqing Medical University, Chongqing, China” it should be “Department of Dermatology, Children’s Hospital of Chongqing Medical University, National Clinical Research Center for Child Health and Disorders, Ministry of Education Key Laboratory of Child Development and Disorders, Chongqing Key Laboratory of Child Infection and Immunity, Chongqing, China”.

In the published article, there was an error in affiliation 4. Instead of “AMITA Health Saini Joseph Hospital Chicago, University of lllinois at Chicago, Chicago, IL, United States” it should be “AMITA Health Saint Joseph Hospital Chicago, University of Illinois at Chicago, Chicago, IL, United States”.

The authors apologize for these errors and state that these do not change the scientific conclusions of the article in any way. The original article has been updated.

